# Cathepsin H increases the risk of diabetic retinopathy: evidence from Mendelian randomization and bioinformatic analysis

**DOI:** 10.1186/s13098-025-01829-y

**Published:** 2025-07-04

**Authors:** Xiudao Song, Jin Ma, Ting Zou, Rong Xin Lu, Wei Ji, Fei Huang, Meiqi Yin

**Affiliations:** 1 Chinese Medicine Technology Transfer Center, Suzhou TCM Hospital Affiliated to Nanjing University of Chinese Medicine, Suzhou, Jiangsu China; 2 State Key Laboratory of Technologies for Chinese Medicine Pharmaceutical Process Control and Intelligent Manufacture, Nanjing University of Chinese Medicine, Nanjing, Jiangsu China; 3 Department of Pharmacy, Children’s Hospital of Soochow University, Jiangsu Suzhou, China; 4 Central Laboratory, Suzhou TCM Hospital, Nanjing University of Chinese Medicine, Suzhou, China; 5 Department of pharmacy, Suzhou TCM Hospital Affiliated to Nanjing University of Chinese Medicine, Jiangsu Suzhou, China; 6 Division of Rheumatology, The Affiliated Hospital of Nanjing University of Chinese Medicine, Nanjing, Jiangsu China; 7 Department of Endocrinology, Suzhou TCM Hospital Affiliated to Nanjing, University of Chinese Medicine, NO.18, Yang Su Road, Suzhou, Jiangsu China

**Keywords:** Mendelian randomization, Bioinformatic analysis, Cathepsins, Diabetic complications, Genome-wide association, Causal relationship

## Abstract

**Background:**

While lysosomal cathepsins are crucial in cellular homeostasis and may contribute to diabetic chronic complications, their precise causal relationships remain insufficiently characterized.

**Methods:**

This study employed comprehensive multidimensional Mendelian randomization (MR) analyses to investigate potential causal associations between nine cathepsins (B, E, F, G, H, L2, O, S, and Z) and six diabetic chronic complications encompassing both microvascular (nephropathy, retinopathy, proliferative retinopathy, maculopathy, neuropathy) and macrovascular manifestations (peripheral angiopathy). The analytical framework incorporated generalized summary-data-based MR (GSMR), univariable MR, multivariable MR, summary-data-based MR (SMR), and cis-expression quantitative trait locus (cis-eQTL) MR approaches, utilizing data derived from publicly available genome-wide association studies (GWAS). To further characterize the biological relevance of identified cathepsins, we conducted multi-omics investigations including gene set enrichment analysis, CIBERSORT, single-cell RNA sequencing analysis to explore the expression level, immune infiltration and biological function of identified cathepsins. Furthermore, we performed mediation analyses to assess whether immune cells potentially mediate the causal pathways linking identified cathepsins to diabetic complications.

**Results:**

Both GSMR and univariable MR showed that Cathepsin H levels were causally associated with increased risks of overall diabetic retinopathy (DR), proliferative diabetic retinopathy (PDR), and diabetic maculopathy, validated by SMR and cis-eQTL MR analyses. Multivariable MR analysis further confirmed the independent causal role of Cathepsin H in PDR {inverse variance weighted (IVW): *p* = 0.003, OR = 1.054, 95% CI = 1.017–1.093} and diabetic maculopathy (IVW: *p* = 0.022, OR = 1.068, 95% CI = 1.009–1.130). Sensitivity analyses indicated no significant heterogeneity or pleiotropy for any of these cause associations. Bioinformatic analyses revealed upregulated Cathepsin H expression in DR patients, enriched in immune-inflammatory pathways. Single-cell RNA sequencing further highlighted its specific overexpression in NK cells of DR. No mediation by immunophenotypes was observed.

**Conclusions:**

Our integrative approach establishes Cathepsin H as a causal driver for DR and its subtypes, highlighting its potential as a therapeutic target and biomarker.

**Supplementary Information:**

The online version contains supplementary material available at 10.1186/s13098-025-01829-y.

## Background

Chronic complications of diabetes mellitus, encompassing microvascular and macrovascular pathologies, represent a substantial public health burden. These complications severely compromise patients’ quality of life and are associated with elevated risks of disability and mortality [1]. Although clinical manifestations of diabetic complications are well-documented, and therapeutic strategies such as metformin, sodium-glucose co-transporter-2 (SGLT2) inhibitors, and glucagon-like peptide-1 (GLP-1) receptor agonists are employed to mitigate risks or delay disease progression, the variable efficacy of these interventions underscores critical gaps in understanding their molecular pathogenesis [2]. Elucidating the underlying etiological mechanisms driving these complications remains an urgent priority in diabetes research.

Emerging evidence implicates lysosomal dysfunction in the pathogenesis of diabetes and its complications [3]. Cathepsins, a family of proteolytic enzymes critical for maintaining cellular homeostasis. The 15 known human cathepsins are classified into three functional categories based on their catalytic activity: aspartyl cathepsins (D, E), cysteine cathepsins (B, C, F, H, K, L, O, S, V, X, W, Z), and serine cathepsins (A, G) [4]. Functioning primarily as endopeptidases within lysosomal compartments, these enzymatic participate in proteostasis, cellular metabolism, autophagic regulation, and growth factor processing [5]. Beyond their degradative roles, cathepsins modulate pathological processes such as antigen presentation, stress-responsive signaling, extracellular matrix remodeling, and lysosome-dependent programmed cell death [6]. Their pleiotropic functions position cathepsins as key mediators in diabetes-associated pathologies, including microvascular and macrovascular complications [3, 6].

Advanced glycation end products (AGEs), formed via non-enzymatic post-translational modification of amino acids, are strongly associated with chronic diabetic complications. Emerging studies have demonstrated the proteolytic capacity of cathepsins in degrading AGEs and their protein adducts [7, 8]. For instance, murine embryonic fibroblasts exposed to AGEs exhibited upregulated cathepsin L and D enzymatic activity, which attenuated AGE-mediated cytotoxic effects [9]. Notably, accumulating evidence implicates specific cathepsins, including Cathepsin H [10], Cathepsin B [11], and Cathepsin L [12], as significant contributors to the development and progression of diabetic microvascular complications. These enzymes contribute to disease progression in diabetic nephropathy (DN) [11, 12], diabetic retinopathy (DR) [10, 13], diabetic neuropathy [14]. Tandem mass tag-based proteomic analysis reveals a novel pathogenic mechanism involving cathepsins in proliferative diabetic retinopathy (PDR) [15]. However, these studies primarily consist of observational studies and clinical trials with a limited sample size. Despite these advancements, the precise causal relationships between specific cathepsins and chronic complications of diabetes remain inadequately defined, highlighting the need for further research to explore these associations and their potential implications for targeted therapeutic interventions.

Mendelian randomization (MR) is a statistical methodology that employs genetic variants associated with exposure traits as instrumental variables (IVs), integrated with genome-wide association study (GWAS) data, to rigorously investigate causal relationships between exposures and outcomes. The random allocation of alleles during gamete formation and their stability throughout lifespan ensures that MR analysis effectively mitigates confounding influences, circumvents reverse causation bias, and provides more reliable causal estimates compared to conventional observational studies [16]. This study systematically investigated causal relationships between nine cathepsins and six chronic diabetic complications by harmonizing large-scale GWAS datasets through a triangulation of methods: bidirectional generalized summary-data-based Mendelian randomization (GSMR), univariable MR, and multivariable MR. To validate findings, summary-data-based Mendelian randomization (SMR) and cis-expression quantitative trait locus (cis-eQTL) MR were employed. Comprehensive bioinformatic analyses were conducted to characterize the expression patterns and functional roles of cathepsins in diabetic complications. Our findings provide novel mechanistic insights into the etiological roles of cathepsins in diabetes-related complications, advancing understanding of their pathophysiological significance.

## Methods

### MR analysis

#### Data sources

This study utilized publicly accessible datasets derived from genome-wide association studies (GWAS). Summary statistics for nine cathepsins were obtained from the INTERVAL study, a cohort comprising 3,301 European participants [17]. These GWAS data are publicly accessible through the MRC Integrative Epidemiology Unit (IEU) GWAS database (https://gwas.mrcieu.ac.uk). Summary statistics for six diabetic complications were acquired from the FinnGen Biobank (https://www.finngen.fi/en), with case numbers ranging from 2,514 (diabetic peripheral angiopathy) to 10,413 (diabetic retinopathy, DR). To ensure population homogeneity and minimize confounding bias, all analyzed data were restricted to European ancestry samples, and these samples were obtained from independent GWAS database. These six diabetic complications included diabetic microvascular complications (DN, DR, PDR, diabetic maculopathy, and diabetic neuropathy) and a diabetic macrovascular complication (diabetic peripheral angiopathy). cis-eQTL summary data were retrieved from the eQTLgen consortium. Detailed descriptions of the GWAS datasets employed in the MR analysis are provided in Table 1.

**Table 1:** Basic information about the datasets included in MR analyses

Trait name	Data sources	Ethnicity/PMID	Sample size (Cases/controls)	IEU Trait ID
Cathepsin B	The INTERVAL study	European/ 29,875,488	3,301	prot-a-718
Cathepsin E	The INTERVAL study	European/ 29,875,488	3,301	prot-a-720
Cathepsin F	The INTERVAL study	European/ 29,875,488	3,301	prot-a-722
Cathepsin G	The INTERVAL study	European/ 29,875,488	3,301	prot-a-723
Cathepsin H	The INTERVAL study	European/ 29,875,488	3,301	prot-a-725
Cathepsin O	The INTERVAL study	European/ 29,875,488	3,301	prot-a-726
Cathepsin S	The INTERVAL study	European/ 29,875,488	3,301	prot-a-727
Cathepsin L2	The INTERVAL study	European/ 29,875,488	3,301	prot-a-728
Cathepsin Z	The INTERVAL study	European/ 29,875,488	3,301	prot-a-729
Diabetic Nephropathy	FinnGen R9	European	312,650 (4,111/308,539)	-
Diabetic Retinopathy	FinnGen R9	European	319,046 (10,413/308,633)	-
Proliferative diabetic retinopathy	FinnGen R9	European	372,092 (9,511/362,581)	-
Diabetic Maculopathy	FinnGen R9	European	312,119 (3,572/308,547)	-
Diabetic Neuropathy	FinnGen R9	European	274,660 (2,843/271,817)	-
Diabetic peripheral angiopathy	FinnGen R9	European	274,331 (2,514/271,817)	-

#### Study design and instrumental variable (IV) selection

**Fig. 1:**
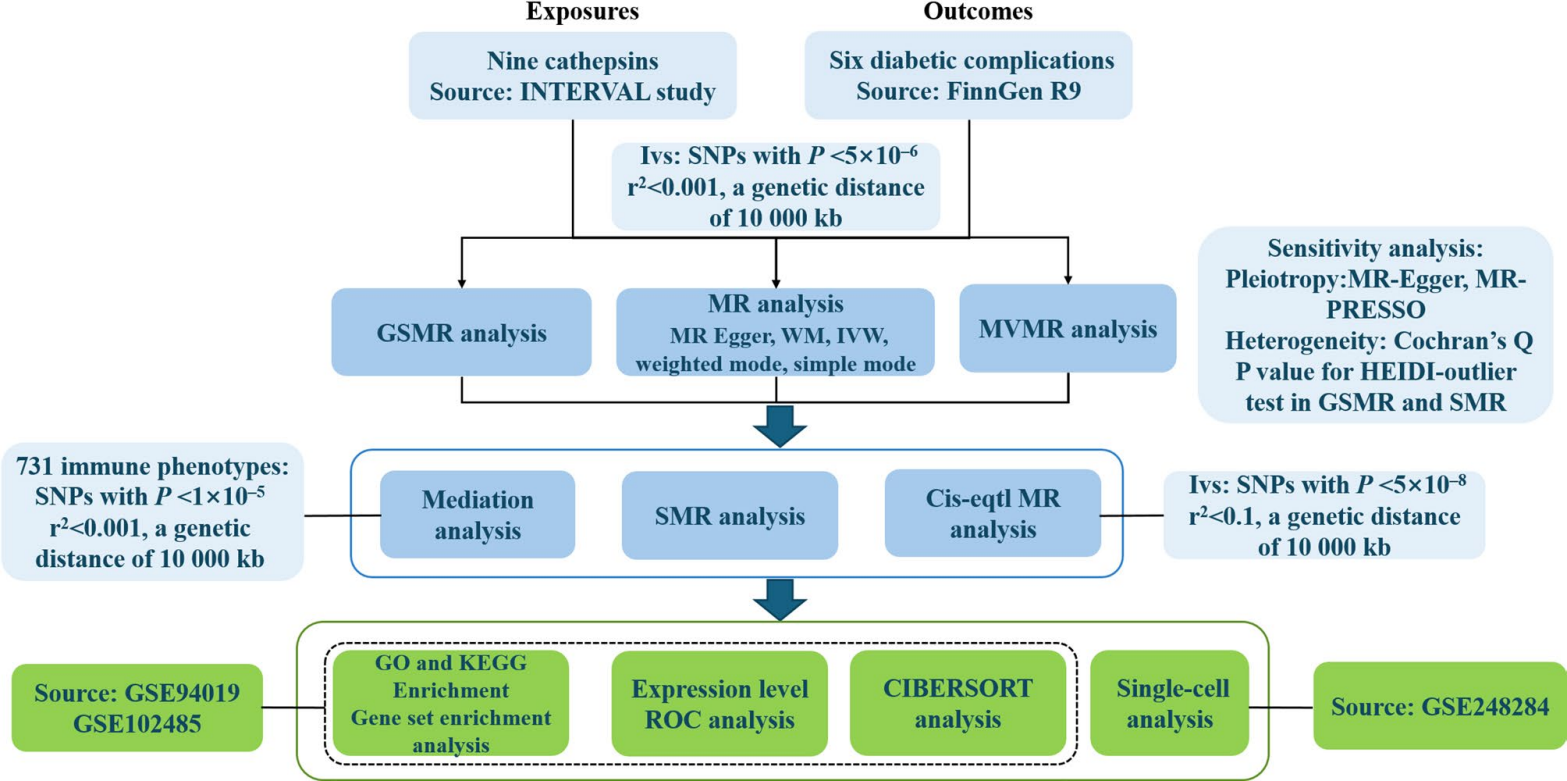
A schematic representation of the study design

IV was rigorously selected based on three fundamental assumptions of MR [18]: correlation assumption, exclusivity assumption, and independence assumption. The first assumption requires a strong link between single nucleotide polymorphisms (SNPs) and the exposure variable. First, a genome-wide significance threshold of *P* < 5 × 10^− 6^ (this value was established in line with the limitation of the sample size) was applied for cathepsins. In reversed MR analysis, SNPs passing genome-wide significance level *P* < 5 × 10^− 8^ were included as IVs for diabetic complications, except for diabetic peripheral angiopathy, for which significance threshold of *P* < 5 × 10^− 6^ was set to address limited SNP availability. Additionally, to remove linkage disequilibrium (LD) [19], an r^2^ < 0.001 for LD and a clump distance of less than 10,000 kb were applied [20]. Second, the selected SNPs had no associations with confounding factors that might affect the exposure-outcome relationship. Finally, the SNPs were confirmed to affect the outcome only through exposure factors. Specifically, the IV could not be extracted from the DR dataset during reverse MR analysis using this method. The selected IVs were also assessed for weak IV bias by calculating the F-test statistic [21]. If F > 10, weak IV bias was considered absent. Additionally, for cis-expression quantitative trait loci (cis-eQTLs), stricter criteria were enforced: *P* < 5 × 10^− 8^, r² < 0.1, and clumping distance < 10,000 kb. Detailed SNP information is available in Supplementary Table 1. A schematic representation of the study design was shown in Fig. 1.

#### Generalized summary Mendelian randomization (GSMR) analysis

We performed bidirectional GSMR utilizing the *gsmr2* package. This approach rigorously accounts for horizontal pleiotropy by excluding SNPs identified as outliers via the heterogeneity in dependent instruments (HEIDI)-outlier test [22]. The HEIDI-outlier approach was applied to remove instruments with strong putative pleiotropic effects, and we specified 0.01 as the *P* value threshold for the HEIDI-outlier filtering analysis [22].

#### MR analysis and sensitivity analyses

Bidirectional causal relationships between cathepsins and diabetic complications were investigated using the TwoSampleMR package (v0.5.7). The inverse variance weighted (IVW) method was selected as the primary estimator due to its efficiency under valid instrumental variable assumptions [23], and results were considered nominally significant at *P* < 0.05. To ensure the robustness of the MR results, MR-Egger, weighted median (WM), weighted mode, and simple mode estimators were used as complementary approaches. Cochran’s Q statistic was calculated using IVW and MR-Egger methods. A random-effects model was applied when *P* < 0.05 (significant heterogeneity); otherwise, a fixed-effects model was used [24]. We also used MR-PRESSO Global Test to further assess and correct for horizontal pleiotropy by identifying and removing outliers (*P* < 0.05 indicating pleiotropic bias). To ensure the reliability of the results, a leave-one-out analysis was performed to exclude SNPs that might have potentially extreme effects. Multivariable MR (MVMR) was implemented to adjust for confounding by simultaneously modeling multiple cathepsins, thereby estimating the direct causal effects of each exposure on the outcomes. All analyses were conducted using R software 4.3.1.

#### Summary data-based Mendelian randomization (SMR) analysis

To investigate the relationships between genes, proteins, and disease, we conducted cis-eQTL SMR and heterogeneity in HEIDI analyses [25]. These analyses leveraged two independent blood-derived eQTL datasets, Genotype-Tissue Expression Project (GTEx, V8) and eQTLgen consortium dataset.

#### Mediation analysis

We conducted a MR mediation analysis to investigate potential mediating effects of immune cells in the causal pathway between Cathepsins and DR. GWAS summary statistics for immune traits are publicly available in the GWAS catalog (accession numbers: GCST90001391-GCST90002121) [26]. The original GWAS analyzed data from 3,757 European individuals, encompassing 731 immune phenotypes classified into four trait types (AC, MFI, MP, RC) and seven panels (B cell, cDC, T cell maturation stages, monocyte, myeloid cell, TBNK, and Treg). First, we evaluated the causal effects of immune cells on DR, with a correlation threshold of 1 × 10^− 5^ for SNPs significantly associated with immune traits. Then we explored whether Cathepsins had a causal effect on immune cells associated with DR, and if so, we would explore whether immune cells were the mediation factors in the pathway from Cathepsins to DR. The Steiger test was applied to address potential issues of reverse causality [27].

### Bioinformatic analysis

#### Data collection

DR-related high-throughput sequencing datasets GSE94019 [28] and GSE102485 [29] were obtained from the Gene Expression Omnibus (GEO) database. The GSE94019 dataset comprises retinal tissue samples from nine patients with PDR and four healthy controls. The GSE102485 dataset includes retinal tissue samples from 21 patients with PDR and three healthy controls.

#### Functional enrichment analysis

Gene Ontology (GO) enrichment analysis encompasses three primary categories: biological processes (BP), cellular components (CC), and molecular functions (MF). GO functional annotation and Kyoto Encyclopedia of Genes and Genomes (KEGG) enrichment analysis were performed using the GOplot (v1.0.2), org.Hs.eg.db (v3.10.0), along with clusterProfiler (v3.14.3) package. Statistical significance was determined using adjusted *P*-value threshold of < 0.05.

#### Gene set enrichment analysis (GSEA)

To delineate the biological roles of Cathepsin H in DR progression, PDR patients were stratified into high-expression and low-expression subgroups based on the median expression level of Cathepsin H, and GSEA was subsequently performed using clusterProfiler[4.4.4] and The Molecular Signatures Database Collections. Statistical significance was defined as adjusted *P*-value < 0.05. The results were visualized using bubble charts.

### CIBERSORT analysis

Transcriptomic profiles of retinal tissues from PDR patients and healthy controls in the GSE94019 dataset were analyzed using the CIBERSORT algorithm to estimate the relative infiltration levels of 22 immune cell subtypes. Differential infiltration abundance between the two groups was systematically evaluated, followed by correlation analysis to investigate potential associations between immune cell subtype proportions and gene expression.

### Analysis of DR single-cell datasets

DR-related single-cell RNA sequencing dataset GSE248284 including three patients with DR and three healthy controls from the GEO database was obtained and analysis. Dimensionality reduction and clustering analysis of the datasets were presented using the t-distributed stochastic neighbor embedding (t-SNE) plot, and the distribution of gene expression in identified cell types was displayed.

## Results

### Bidirectional GSMR analysis between cathepsins and diabetic chronic complications

**Fig. 2:**
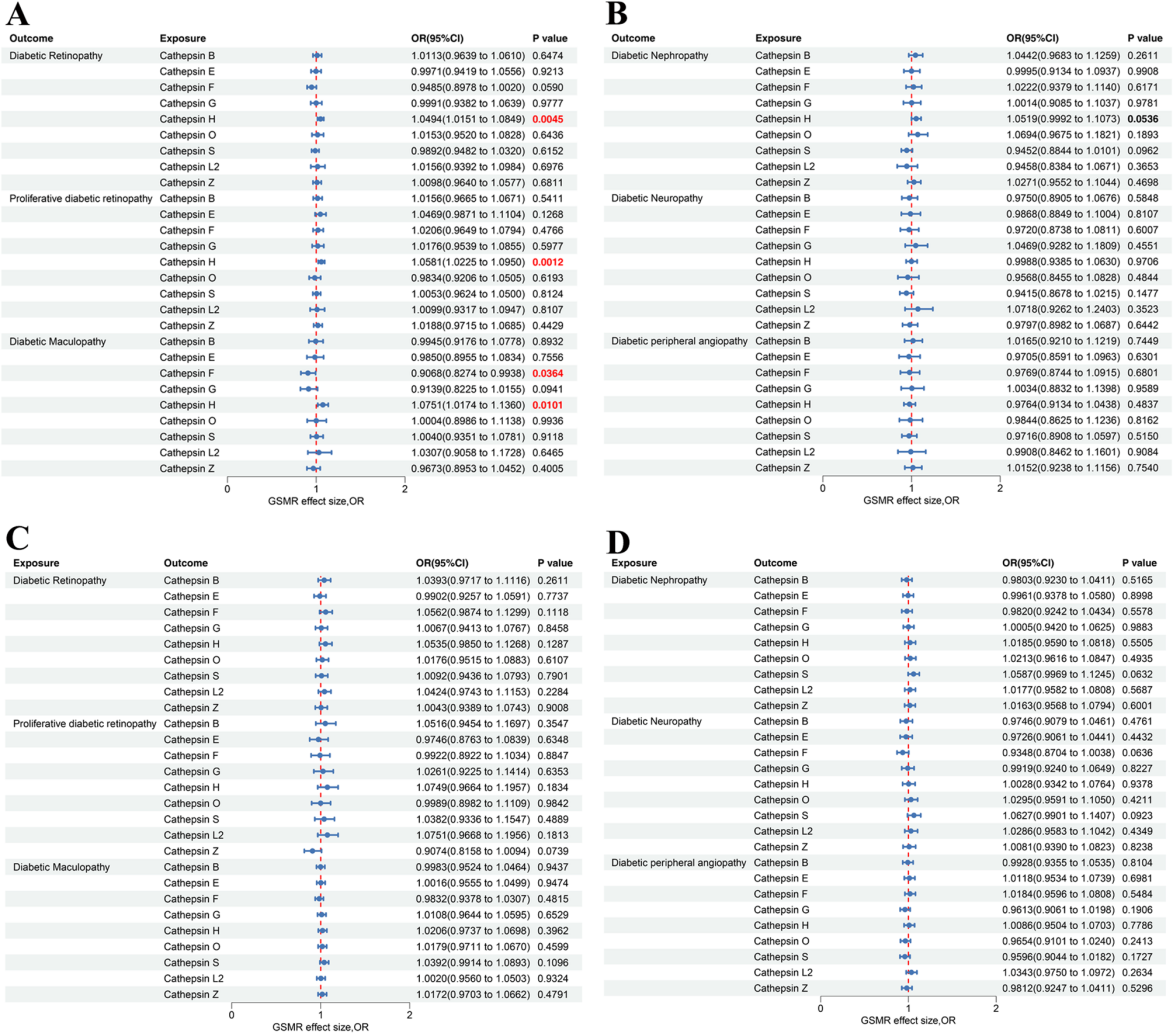
Forest plot for the bidirectional GSMR analysis between six cathepsins and nine diabetic chronic complications. (**A**, **B**) GSMR effect of cathepsins on diabetic chronic complications. (**C**, **D**) GSMR effect of diabetic chronic complications on cathepsins

Using GSMR, we systematically evaluated the causal effect of nine cathepsins (B, E, F, G, H, L2, O, S, Z) on six diabetic chronic complications risk (Fig. 2). Forward-direction GSMR analysis showed that Cathepsin H was positively associated with DR (OR = 1.0494, 95% CI = 1.0151–1.0849, *P* = 4.5 × 10^− 3^), proliferative DR subtype PDR (OR = 1.0581, 95% CI = 1.0225–1.0950, *P* = 1.2 × 10^− 3^), and diabetic maculopathy (OR = 1.0751, 95% CI = 1.0174–1.1360, *P* = 0.0101). Cathepsin F showed a protective effect against diabetic maculopathy (OR = 0.9068, 95% CI = 0.8274–0.9938, *P* = 0.0364) (Fig. 2A). No significant causal effects were observed for other cathepsins across diabetic nephropathy, neuropathy, or peripheral angiopathy endpoints (Fig. 2B). The reverse-direction GSMR analysis revealed no statistically significant effects of diabetic complications on cathepsins (Fig. 2C and D).

### Univariable MR analysis between cathepsins and diabetic chronic complications

**Fig. 3:**
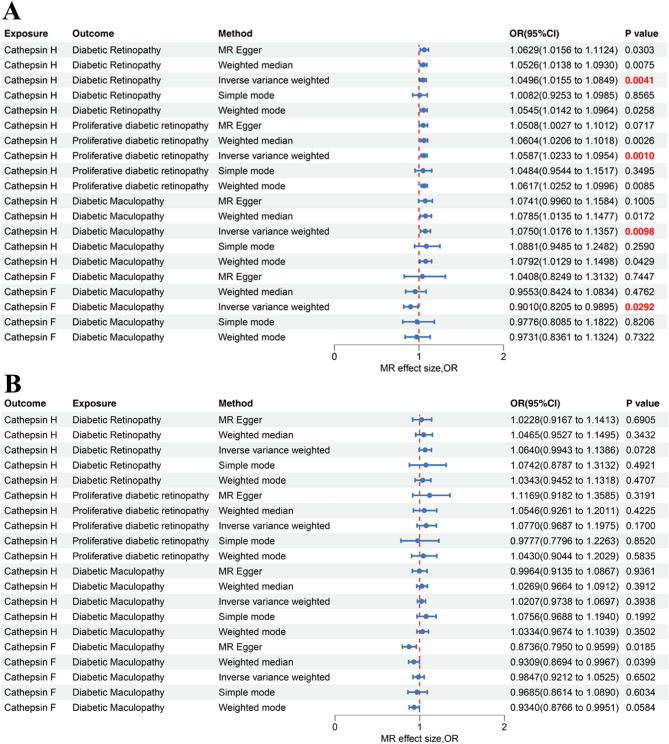
Forest plot of univariable Mendelian randomization analysis between six cathepsins and nine diabetic chronic complications. (**A**) MR effect of Cathepsin H/F on the risk of diabetic retinopathy, proliferative diabetic retinopathy and diabetic maculopathy. (**B**) MR effect of diabetic retinopathy, proliferative diabetic retinopathy and diabetic maculopathy on Cathepsin H/F

Two-sample MR employing IVW primary analysis validated the GSMR findings and explored sensitivity analysis. High levels of Cathepsin H increased the risk of DR (OR = 1.0496, 95% CI = 1.0155–1.0849, *P* = 4.1 × 10^− 3^), and other two subtypes of DR, including PDR (OR = 1.0587, 95% CI = 1.0233–1.0954, *P* = 1.0 × 10^− 3^) and diabetic maculopathy (OR = 1.075, 95% CI = 1.0176–1.1357, *P* = 9.8 × 10^− 3^), as shown in Fig. 3A. The WM method and the weighted model method further confirmed a significant association between higher Cathepsin H levels and an increased risk of DR, PDR and diabetic maculopathy (Fig. 3). Additionally, increased levels of Cathepsin F decrease the risk of diabetic maculopathy (OR = 0.9010, 95% CI = 0.8205–0.9895, *P* = 0.0292). Interestingly, the IVW-MR analysis also showed that high levels of Cathepsin H were associated with an increased risk of DN (OR = 1.0533, 95% CI = 1.0008–1.1087, *P* = 0.04639). Furthermore, neither the MR-Egger intercept nor the MR-PRESSO global test provided evidence of directional pleiotropy for the above causal associations, and Cochran’s Q (MR-Egger and IVW) indicated that there was no significant heterogeneity (*P*>0.05; Supplementary Table 2). The leave-one-out analysis results demonstrate the robustness of these causal associations, as shown in Supplementary data 1. Therefore, the IVW-MR further confirmed the GSMR results for Cathepsin H on DR, PDR and diabetic maculopathy, and cathepsin F on diabetic maculopathy. A reverse MR analysis was also performed using the six diabetic chronic complications as exposures and the nine cathepsins as outcomes, and the results showed no statistically significant effects of diabetic complications on cathepsins (Fig. 2B; Supplementary Table 2).

### Multivariable MR analysis between cathepsins and diabetic chronic complications

**Fig. 4:**
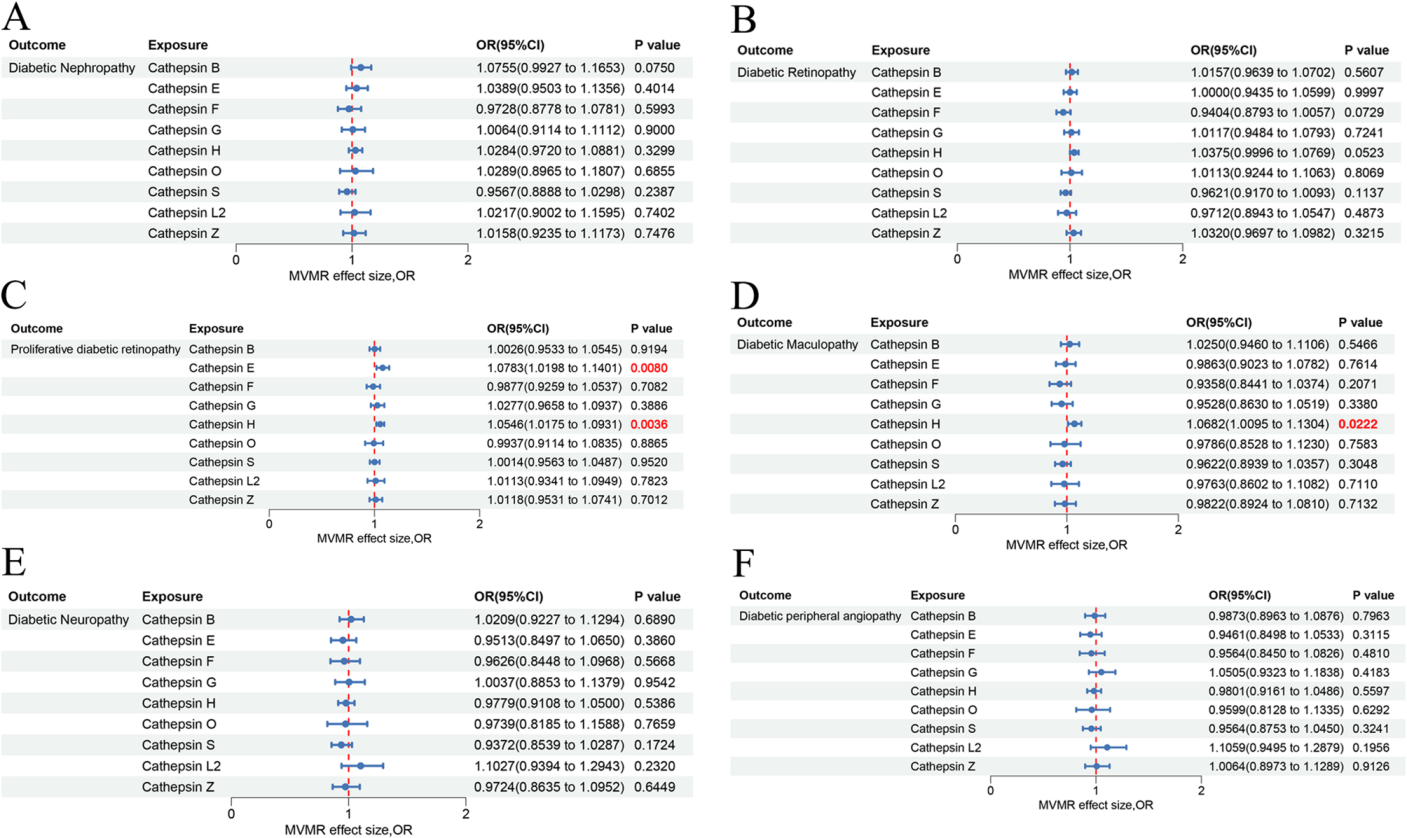
Forest plot of multivariable Mendelian randomization analysis for six cathepsins and nine diabetic chronic complications. We employed the IVW method to investigate the causal associations between nine cathepsins (Cathepsin B, E, F, G, H, L2, O, S, and Z) and diabetic nephropathy (**A**), diabetic retinopathy (**B**), proliferative diabetic retinopathy (**C**), diabetic maculopathy (**D**), and diabetic neuropathy (**E**), and diabetic peripheral angiopathy (**F**)

Multivariable MR analysis was performed to disentangle independent causal effects of nine cathepsins on diabetic complications. The MVMR results showed that even after adjusting for other types of cathepsins, the association between Cathepsin H and PDR (OR = 1.0546, 95% CI = 1.0175–1.0931, *P* = 3.6 × 10^− 3^) (Fig. 4C) and diabetic maculopathy (OR = 1.0682, 95% CI = 1.0095–1.1304, *P* = 0.0222) (Fig. 4D) still showed a significant positive causal relationship. Interestingly, Cathepsin E exhibited previously obscured association with PDR (OR = 1.0783, 95% CI = 1.0198–1.1401, *P* = 8.0 × 10^− 3^) (Fig. 4C). However, no statistically significant causal association was observed between Cathepsin H and DN, Cathepsin H and DR, or Cathepsin F and diabetic maculopathy, after adjusting for other types of cathepsins (Supplementary Table 3).

### Cis-eQTL MR and summary Mendelian randomization (SMR) analysis of cathepsin H

To validate the causal effect of Cathepsin H on DR, PDR, and diabetic maculopathy risks, we obtained cis-eQTL data from the eQTLGen Consortium. MR analysis demonstrated significant associations between Cathepsin H cis-eQTLs and increased risks of DR (OR = 1.0622, 95% CI = 1.0296–1.0958, *P* = 1.0 × 10^− 4^), PDR (OR = 1.0671, 95% CI = 1.0328–1.1026, *P* = 9.99 × 10^− 5^), and diabetic maculopathy (OR = 1.1273, 95% CI = 1.0654–1.1928, *P* = 3.17 × 10^− 5^). The MR-Egger intercept and the MR-PRESSO global test did not show significant horizontal pleiotropy, and Cochran’s Q test (MR-Egger and IVW) did not detect any heterogeneity. We further used SMR analysis to validate the relationship among the cis-eQTL of Cathepsin H and DR, PDR, and diabetic maculopathy. SMR analysis confirmed these relationships using eQTLGen data, showing significant associations between Cathepsin H variants and DR risk (OR = 1.0715, 95% CI: 1.0220–1.1235, *P* = 4.2 × 10^− 3^, P_HEIDI = 0.8443), PDR (OR = 1.0809, 95% CI: 1.0295–1.1349, *P* = 1.8 × 10^− 3^, P_HEIDI = 0.8063), and diabetic maculopathy (OR = 1.0996, 95% CI: 1.0168–1.1893, *P* = 0.0175, P_HEIDI = 0.5574). These results were replicated using GTEx cis-eQTL data (Table 2). The details of MR and SMR results, the instrumental SNPs for the Cathepsin H, and heterogeneity and pleiotropy assessments can be found in Supplementary Table 4.

**Table 2:** Results of SMR analysis conducted in this study

Consortium	Outcome	p_SMR	p_HEIDI
eQTLGen	Diabetic retinopathy	0.0042	0.8444
eQTLGen	Diabetic maculopathy	0.0175	0.5574
eQTLGen	Proliferative diabetic retinopathy	0.0018	0.8063
GTEx_V8_cis_eqtl_Whole_Blood.lite	Diabetic retinopathy	0.0086	0.4030
GTEx_V8_cis_eqtl_Whole_Blood.lite	Diabetic maculopathy	0.0222	0.3558
GTEx_V8_cis_eqtl_Whole_Blood.lite	Proliferative diabetic retinopathy	0.0037	0.3356

### Expression and function analysis of cathepsin H

**Fig. 5:**
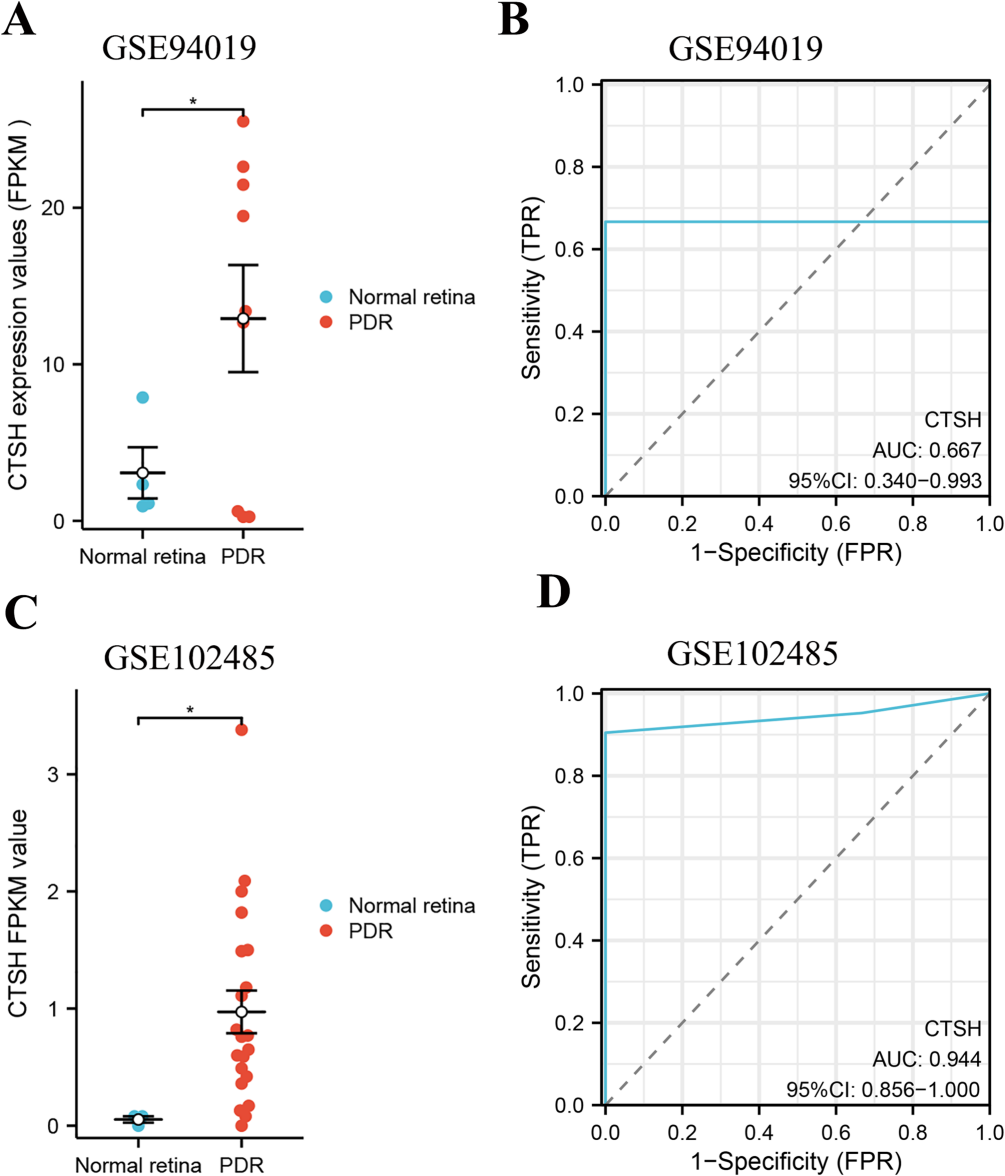
Expression levels and ROC curves of Cathepsin H in PDR. The expression levels of genes (**A**) and ROC curves (**B**) of Cathepsin H in the GSE94019 dataset. The expression levels of genes (**C**) and ROC curves (**D**) of Cathepsin H in the GSE102485 dataset. ^*^P < 0.05; Cathepsin H, CTSH

Given MR evidence linking elevated Cathepsin H levels to DR, we investigated its expression patterns in PDR. Two independent datasets (GSE94019 and GSE102485) confirmed significant Cathepsin H upregulation in DR (Fig. 5A and C), consistent with MR findings. The ROC curve analysis shows the diagnostic performance of Cathepsin H in two independent datasets: in the GSE94019 dataset, the AUC was 0.667 (95% CI: 0.340–0.993; Fig. 5B), indicating moderate discriminative ability; whereas in the GSE102485 dataset, the AUC significantly increased to 0.944 (95% CI: 0.856-1.000; Fig. 5D).

**Fig. 6:**
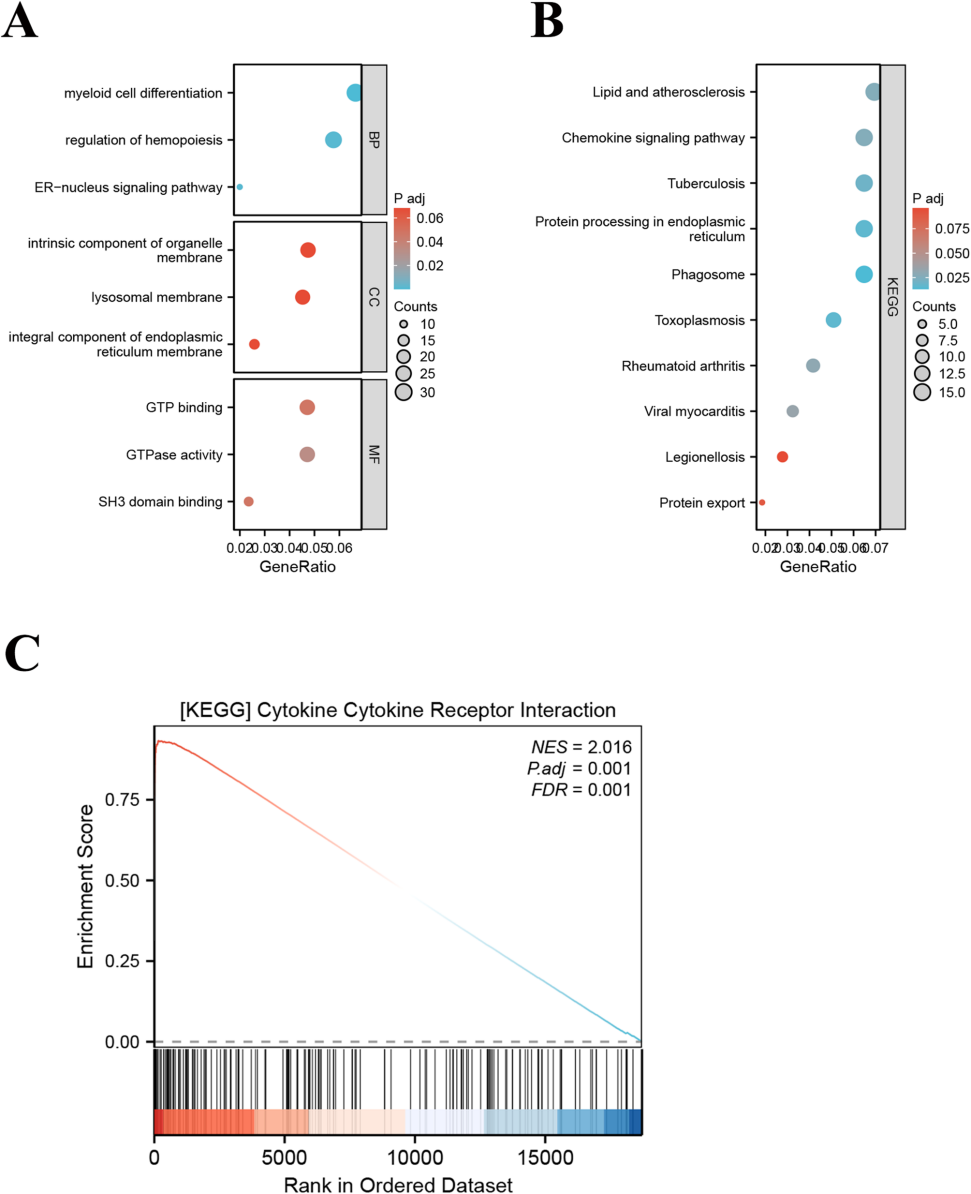
Function analysis of Cathepsin H in PDR. Bubble plot of enriched GO (**A**) and KEGG terms of 577 differentially expressed genes. Y-axis: name of GO items; X-axis: percentage of the number of genes assigned to a term among the total number of genes annotated in the network; Bubble size, number of genes assigned to a pathway; Color: enriched -log10 (*P*-value); Red bubble: indicates a greater significance level. (**C**) GSEA analysis of Cathepsin H

Based on the median expression of Cathepsin H, PDR patients from GSE94019 were divided into high-expression and low-expression groups, and 577 differentially expressed genes were identified, applying the criteria of|logFC| >1 and a *P*-value < 0.05 (Supplementary Table 5). We performed GO enrichment and KEGG enrichment analysis on these 577 differentially expressed genes. GO analysis revealed enrichment in myeloid cell differentiation, regulation of hemopoiesis, and ER-nucleus signaling pathway (Fig. 6A). KEGG analysis identified involvement in Phagosome, Protein processing in endoplasmic reticulum, Toxoplasmosis, Tuberculosis, and Chemokine signaling pathway (Fig. 6B). GSEA highlighted significant enrichment in cytokine-cytokine receptor interactions (Fig. 6C). Collectively, these results suggest that Cathepsin H’s role in immune-inflammatory pathways.

### Immune landscape of cathepsin H

**Fig. 7:**
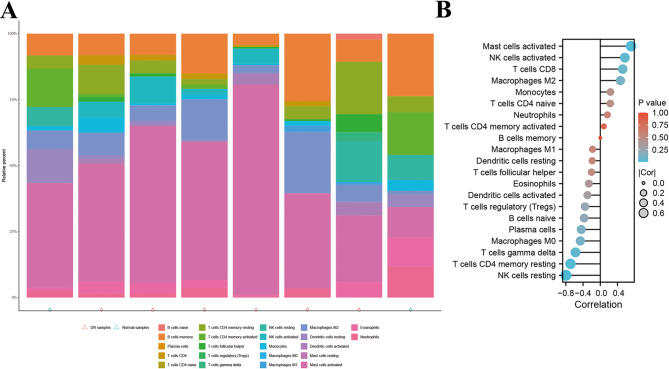
Immune landscape of Cathepsin H in PDR. (**A**) The composition of the 22 immunocytes on DR and normal samples identified via the CIBERSORT arithmetic. (**B**) The spearman correlation analysis between Cathepsin H and immune cells

The CIBERSORT algorithm was employed to compare immune cell infiltration patterns between DR and control samples. Its composition in PDR and normal samples was displayed in Fig. 7A, offering insights into the immune cell landscape associated with PDR. Thus, we further examined the correlation between Cathepsin H expression and immune cells. As shown in Fig. 7B, spearman correlation analysis showed that Cathepsin H showed a positively significant relationship with NK cells activated (*r* = 0.57) and Mast cells activated (*r* = 0.71), while having a negative correlation with T cells CD4 memory resting (*r*=-0.69), T cells gamma delta (*r*=-0.58), and NK cells resting (*r*=-0.80). These findings suggest that Cathepsin H plays a significant role in modulating the behavior and abundance of specific immune cell populations in PDR.

### Single-cell transcriptomic profiling of cathepsin H in peripheral blood mononuclear cells (PBMC)

**Fig. 8:**
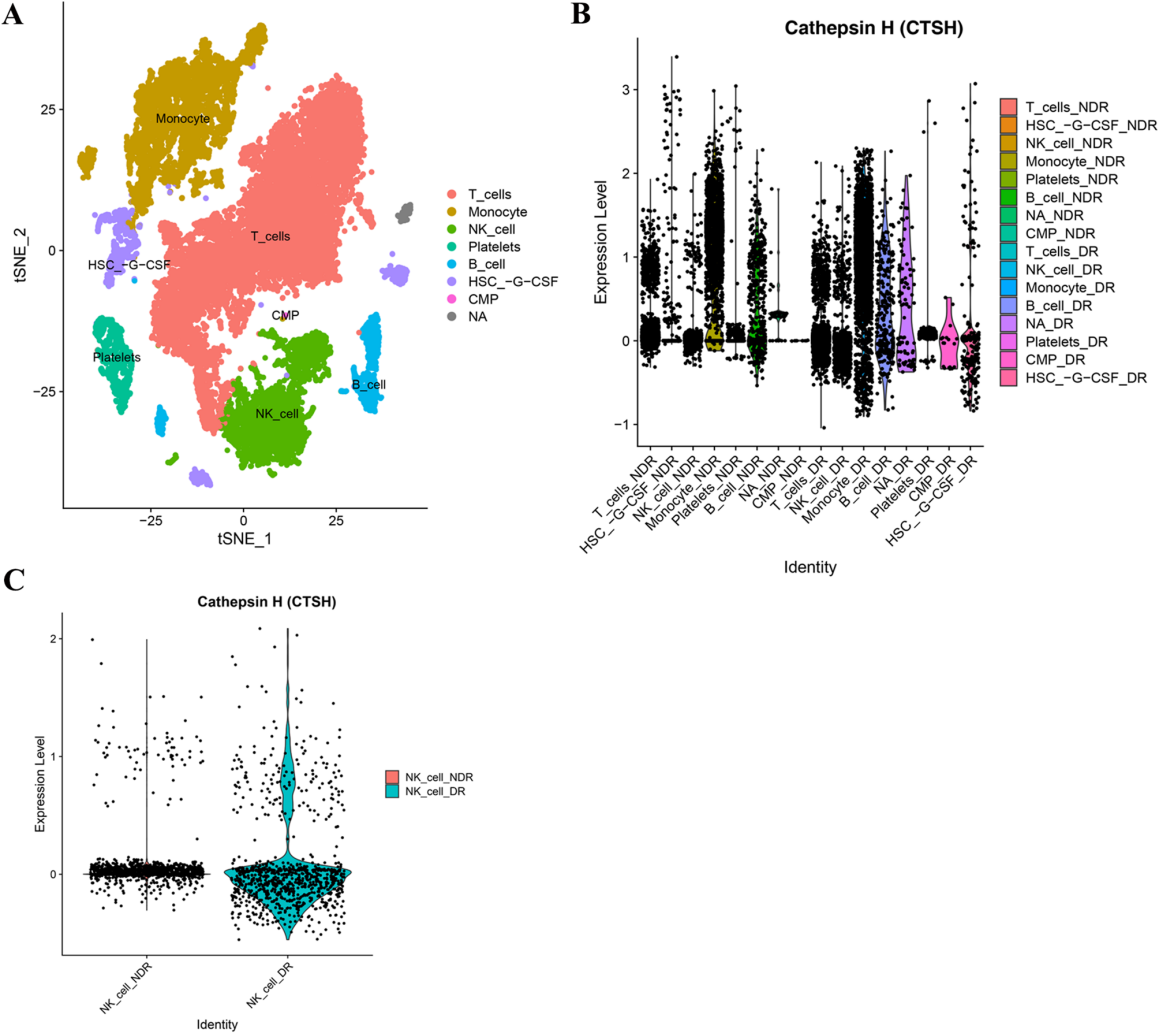
Gene CTSH expression analysis in the single-cell transcriptome dataset GSE248284. (**A**) The t-distributed stochastic neighbor embedding (t-SNE) plot of the seven identified main cell types among single-cell transcriptome dataset GSE248284. (**B**) Correlation of CTSH with seven types of main cell types between the NDR and DR in single cell transcriptome data

We obtained single-cell RNA-seq dataset GSE248284 from the NCBI GEO database and conducted comprehensive preprocessing including quality control, normalization, and highly-variable gene screening. After dimensional reduction through principal component analysis, cellular clusters were visualized using t-SNE projection (Fig. 8A). Notably, spatial expression mapping revealed distinct Cathepsin H distribution patterns across PBMC subpopulations (Fig. 8B). Comparative analysis demonstrated significant Cathepsin H upregulation in NK cells of DR samples (average log2FC = 1.32, adjusted *P* value = 1.02 × 10^− 25^), as shown in Fig. 8C.

### Mediation analysis

Given Cathepsin H’s potential immune-modulatory role in DR, it seemed that immune cells might mediate its effects on DR pathogenesis. Using two-sample MR analysis, we investigated immunophenotypic causal effects across DR spectrum disorders (DR, PDR, diabetic maculopathy). For DR, a total of 40 immunophenotypes were identified with a *P*-value threshold of < 0.05 used IVW-MR analysis, including nine B cell types, five cDC types, five Maturation stages of T cell types, five Monocyte types, three Myeloid cell types, six TBNK types, and seven Treg types. Among identified 40 immunophenotypes, the significant immunophenotype was HLA DR on DC (*P* = 1.46 × 10^− 6^, OR = 1.4414, 95% CI = 1.2421–1.6727). For PDR, a total of 59 immunophenotypes were identified with a *P*-value threshold of < 0.05 used IVW-MR analysis, including 27 B cell types, four cDC types, six Maturation stages of T cell types, three Monocyte types, three Myeloid cell types, eight TBNK types, and eight Treg types. Among identified 59 immunophenotypes, the significant immunophenotype was HLA DR on DC (*P* = 8.47 × 10^− 7^, OR = 1.3092, 95% CI = 1.1761–1.4574). For diabetic maculopathy, a total of 36 immunophenotypes were identified with a *P*-value threshold of < 0.05 used IVW-MR analysis, including three B cell types, nine cDC types, four Maturation stages of T cell types, four Monocyte types, two Myeloid cell types, six TBNK types, and eight Treg types. Among identified 36 immunophenotypes, the significant immunophenotype was HLA DR on DC (*P* = 2.96 × 10^− 7^, OR = 1.6664, 95% CI = 1.3708–2.0257). One of the requirements for mediating effect is that Cathepsin H was significantly associated with immunophenotypes. However, our results revealed that there were no causal relationships between Cathepsin H and immune phenotypes in DR pathogenesis (Supplementary Table 6), which suggested that immune cells did not act as a mediator in the pathway from Cathepsin H and DR, PDR, and diabetic maculopathy.

## Discussion

The pathogenesis of diabetic microvascular and macrovascular complications involves intricate proteolytic cascades, among which cathepsins - lysosomal proteases governing cellular homeostasis through protein degradation, autophagy, and apoptosis - have emerged as pivotal regulators [30]. While prior studies suggested associations between cathepsin dysregulation and diabetic complications [10, 11, 14, 31], our study establishes the first multidimensional genetic evidence for the causal relationships between nine cathepsins and six diabetic chronic complications. Our GSMR and IVW-MR analyses robustly identified Cathepsin H as a novel causal risk factor for DR and its severe subtypes PDR and diabetic maculopathy. Crucially, MVMR accounting for protease family collinearity confirmed the independent causal role of Cathepsin H in PDR and diabetic maculopathy. Complementary analyses, including cis-eQTL MR and SMR, further validated the causal roles of Cathepsin H in DR, PDR, and diabetic maculopathy. The upregulation Cathepsin H expression in PDR patients was also identified by bioinformatic analysis. By synergizing GSMR, MVMR, IVW-MR and SMR with multi-omics integration, this work not only establishes Cathepsin H as a novel therapeutic target for PDR and diabetic maculopathy but also pioneers a translational paradigm bridging genetic association to clinical intervention.

Cathepsin H, a papain-like cysteine protease synthesized as an inactive zymogen requiring proteolytic activation [32], orchestrates multifaceted biological processes spanning endolysosomal degradation, protease cascade initiation, anti-apoptotic signaling, and MHC class II antigen presentation [33, 34]. Emerging translational evidence implicates Cathepsin H in metabolic disorders, with elevated circulating levels associated with type 1 diabetes mellitus (T1DM) risk [35]. Genetic evidence showed the protective effect of Cathepsin H/rs3825932-T allele against PDR progression [10], and the minor allele T has previously been associated with decreased susceptibility to the development of T1DM, which could explain the protective effect of this allele [36]. By integrating GSMR with multidimensional genetic evidence, this study systematically elucidates the stage-specific pathogenic role of Cathepsin H in DR. Initial GSMR analysis identified Cathepsin H as a risk factor for overall DR, PDR, and diabetic maculopathy, with findings strongly concordant with IVW-MR. Furthermore, the analytical robustness was fortified through HEIDI-outlier filtering (pleiotropic SNPs, pHEIDI < 0.01), MR-PRESSO, and Cochran’s Q heterogeneity testing. This causal gradient was further corroborated by MR analyses identifying plasma and brain-derived Cathepsin H as independent contributors to DR pathogenesis [37]. Crucially, MVMR adjusting for protease family demonstrated substantial attenuation in Cathepsin H-DR association, while preserving robustness in PDR and diabetic maculopathy subgroups, indicating stage-dependent intensification of proteolytic effects. Finally, although MR analysis showed that Cathepsin H as a risk factor for DR had a modest effect size, this sustained low-level risk exposure may significantly increase the disease burden through additive effects.

Emerging evidence underscores the pivotal role of immune dysregulation and inflammatory microenvironment in DR development [38], and Cathepsin H expression level is increased in activated immune cells including dendritic cells, macrophages, and microglia [39], suggesting Cathepsin H-mediated immune modulation in DR progression. Our integrated bioinformatics analyses corroborated these findings: CIBERSORT revealed Cathepsin H-associated immune cell infiltration signature and GSEA identified significant enrichment of Cathepsin H in immune-inflammation pathways. Our single-cell analyses further localized Cathepsin H overexpression specifically to NK cell subsets, suggesting Cathepsin H⁺ NK cells preferentially accumulate in PDR vitreous. Paradoxically, mediation analysis revealed no significant contribution of immune cell or inflammatory cytokines to Cathepsin H-mediated DR progression, suggesting the involvement of Cathepsin H in DR pathogenesis through other regulatory mechanism. Similar to other cysteine cathepsins, Cathepsin H mediates various proteolytic processes, and we thereby have reason to believe that Cathepsin H may process pro-angiogenic factors like VEGF and thereby directly promoting pathological neovascularization in DR. Intriguingly, the pro-angiogenic capacity of Cathepsin H, evidenced by defective pericyte recruitment in Cathepsin H-deficient tumors [40], provides mechanistic plausibility for its role in DR-associated neovascularization. Since Cathepsin D/L is one of the major enzymes involved in intracellular degradation of AGE-modified proteins [9], whether Cathepsin H also may interact with AGE-RAGE axis to involve in DR. Additional, as upregulation of Cathepsin H led to matrix metallopeptidase [41], Cathepsin H may regulate the degradation of retinal vascular basement membranes by modulating matrix metalloproteinase activity. Although Cathepsin B/D have been implicated in hyperglycemia-anti-autophagic and pro-apoptotic effects under PDR [15], Cathepsin H’s specific role in retinal vascular pathology remains uncharacterized. Future investigations should employ retinal organoids or Cathepsin H conditional knockout models to dissect its immunomodulatory and angiogenic functions and assess the impacts on the above signaling pathways.

Our IVW-MR analyses extend the causal implications of cathepsins to DN, revealing novel associations with Cathepsin H. As a microvascular complication affecting ~ 40% of diabetics and the leading global cause of end-stage kidney disease (ESKD) [42], DN pathogenesis may involve Cathepsin H-mediated pathways, given its renal expression in podocytes and proximal tubules [43]. Despite this anatomical plausibility, direct mechanistic links between Cathepsin H and DN remain uncharacterized. Cathepsin F is a cysteine protease known to inhibit angiogenesis [44]. Equally intriguing is the protective role of Cathepsin F against diabetic maculopathy in our GSMR and IVW-MR analysis. For Cathepsin E, an intracellular, hydrolytic aspartic protease that is expressed in cells of the immune and gastrointestinal systems, lymphoid tissues, erythrocytes, and cancer cells [45], MVMR identified a diabetes subtype-independent risk association with PDR, absent in univariable analysis. This discrepancy may be partly explained by the complex interactions between cathepsins and the fact that we did not distinguish whether PDR was due to type 1 or type 2 diabetes. Although increased urinary activities of cathepsins have been observed and proposed as early markers of DN [46, 47], including cathepsins B, D, and K in the urine of T2DM or diabetic kidney disease [47, 48], cathepsin E appears to decrease, showing a significant trend in the macroalbuminuric group of DN patients [49], implying divergent regulatory roles in diabetic complications. These findings collectively underscore the necessity for tissue- and stage-specific profiling of cathepsin activities to disentangle their pleiotropic effects.

Several methodological limitations of this study warrant cautious interpretation. First, our study’s exclusive focus on European ancestry cohorts, while methodologically necessary to ensure genetic homogeneity in the two-sample MR framework, inherently limits the extrapolation of Cathepsin H-DR causal relationships to non-European populations. This constraint may stem from two interlinked factors: (1) The differences in allele frequency across different populations [50] may could substantially alter instrument variable strength in trans-ethnic MR analyses; (2) Ethnic-specific environmental exposures may interact with genetic variants through mechanisms like DNA methylation, thereby modulating Cathepsin H-DR associations. Thus, future research will need to use Asian/African populations to confirm the applicability of causal relationships across different populations. Second, while MR analysis provides theoretical causal evidence at the genetic level, it is required further biological validation through animal models and randomized controlled clinical trials. Third, due to the small number of cases, data on diabetic cardiovascular disease and ischemic stroke were not included, which may have resulted in inadequate power in the analysis of diabetic complications. Fourth, we were unable to differentiate between the complications of type 1 and type 2 diabetes, leaving the applicability of the results to the chronic complications of either type uncertain. Regardless, this study first identified Cathepsin H as a causal risk factor for DR, highlighting its potential as both a diagnostic biomarker and therapeutic target for clinical translation. In terms of diagnosis, the plasma levels of Cathepsin H positively correlate with DR, suggesting its utility as a non-invasive biomarker for early screening and dynamic monitoring of disease progression. For therapeutic development, Cathepsin H -specific inhibitors [51, 52], some of which are currently under development, may hold significant clinical value.

## Conclusions

Our study pioneers the integration of bidirectional MR and multi-omics analyses to systematically establish causal relationships between nine cathepsins and six diabetic chronic complications. Leveraging consortium-level genetic data (eQTLGen, GTEx) and functional validation (GSE94019, GSE102485), we identified Cathepsin H as a key pathogenic driver of PDR and diabetic maculopathy, with robust evidence from GSMR, MVMR, and cis-eQTL-based SMR. Multi-omics analyses spanning genetic variation to expression regulation, immune infiltration, and single-cell levels comprehensively elucidate the biological significance of Cathepsin H in DR. These findings suggests high expression of Cathepsin H is strongly associated with DR and its subtypes (PDR and diabetic maculopathy), positioning it as a candidate biomarker for diagnosis or disease stratification and suggesting targeted inhibition of Cathepsin H may delay DR progression, particularly for patients with PDR and diabetic maculopathy. Further studies are needed to elucidate whether Cathepsin H drives DR by modulation of angiogenic processes or activation of immune-mediated inflammatory cascades.

## Electronic supplementary material

Below is the link to the electronic supplementary material.

**Supplementary Material 1**: **Supplementary data 1**. Leave-one-out analysis between cathepsins and different diabetic chronic complications. **Supplementary Table 1**. Characteristics of SNPs serving as instrumental variables. **Supplementary Table 2**. The results of bidirectional MR analysis between six cathepsins and the risk of nine diabetic chronic complications. **Supplementary Table 3**. Multivariable MR analysis of cathepsins and different diabetic chronic complications. **Supplementary Table 4**. The results of cis-eQTL MR and SMR analysis. **Supplementary Table 5**. 577 differentially expressed genes identified from GSE94019 based on the median expression expression of Cathepsin H. **Supplementary Table 6**. The causal effects of immunophenotypes on DR, PDR, and diabetic maculopathy, and the causal effects of Cathepsin H on immunophenotypes associated with DR, PDR, or diabetic maculopathy.

## Data Availability

The dataset analyzed in this study can be accessed through the following websites: IEU GWAS website (https://gwas.mrcieu.ac.uk), FinnGen (https://www.finngen.fi/en), GWAS Catalog website (https://www.ebi.ac.uk/gwas/) GEO (https://www.ncbi.nlm.nih.gov/geo/), and eQTLGen Consortium (https://www.eqtlgen.org/phase1.html).
